# Supplementary material for: Distinct patterns of activity in individual cortical neurons and local networks in primary somatosensory cortex of mice evoked by square-wave mechanical limb stimulation
Source: PLoS One. 2021 Apr 29;16(4):e0236684. doi: 10.1371/journal.pone.0236684 (PMC8084136; doi:10.1371/journal.pone.0236684)
Supplement: S1 Fig — In this example depicting the stimulus-evoked correlation maps from an example animal in the cHL at 130um depth, the SSIM value and SSIM comparison image display greater similarity for within trials of 0.1s 100Hz (A) and within trials for 1s 100Hz (B). Comparisons between trials of stimuli with different frequency or temporal durations demonstrate less structural similarity and a lower SSIM value (C). To generate the SSIM charts seen in Figs 5 and 6 and 8 trials of each stimulus were compared for SSIM value within and between stimuli as shown in (D) (comparison between 4 trials of each shown here). (DOCX) [file pone.0236684.s001.docx]

**
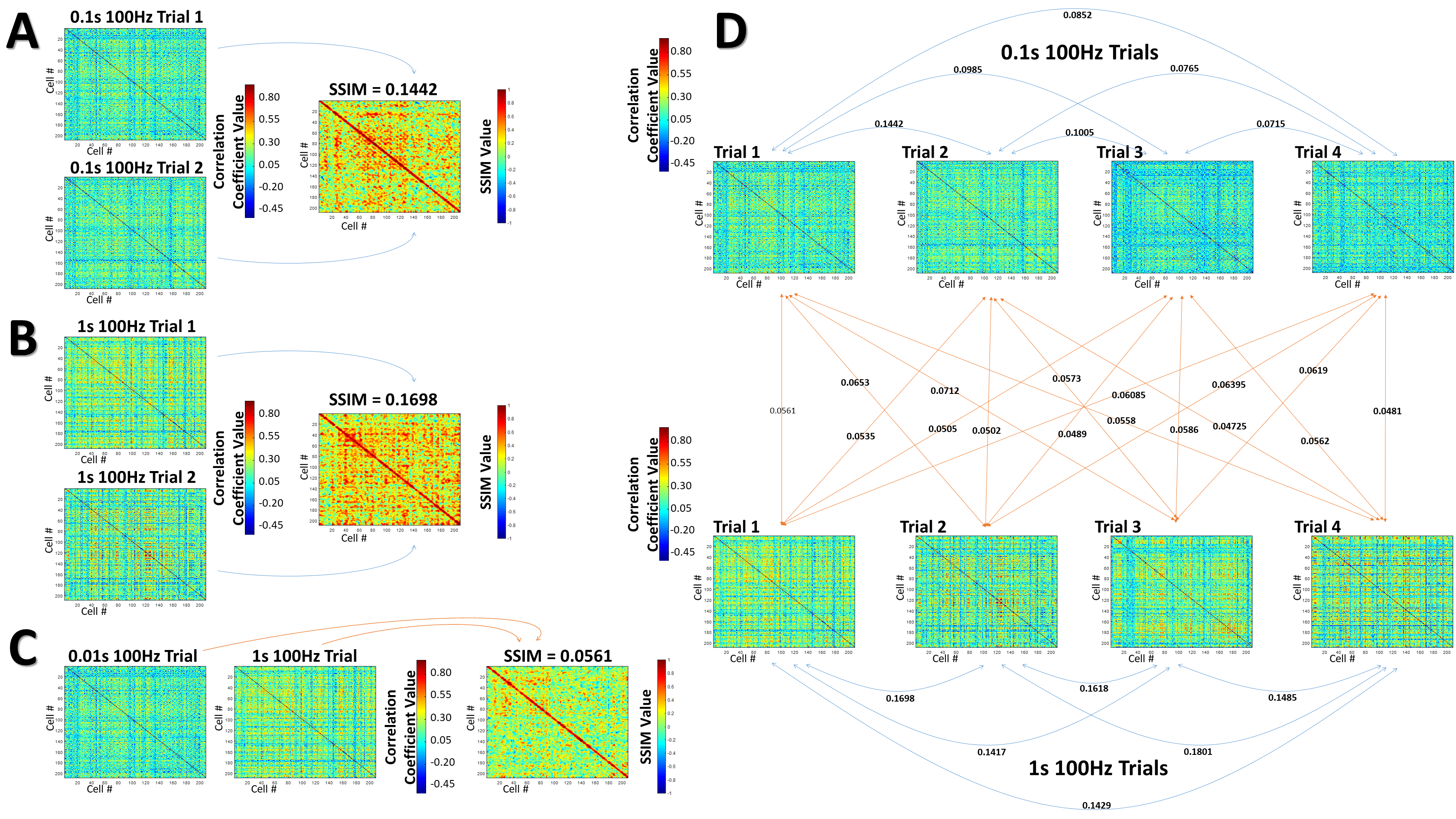
**

**Fig S1. Visual example of the structural similarity (SSIM)-based method for trial-by-trial comparisons of correlational map similarity.** In this example depicting the stimulus-evoked correlation maps from an example animal in the cHL at 130um depth, the SSIM value and SSIM comparison image display greater similarity for within trials of 0.1s 100Hz (***A***) and within trials for 1s 100Hz (***B***). Comparisons between trials of stimuli with different frequency or temporal durations demonstrate less structural similarity and a lower SSIM value (***C***). To generate the SSIM charts seen in Figs. 5&6, 8 trials of each stimulus were compared for SSIM value within and between stimuli as shown in (***D***) (comparison between 4 trials of each shown here).
