# Supplementary material for: Distinct patterns of activity in individual cortical neurons and local networks in primary somatosensory cortex of mice evoked by square-wave mechanical limb stimulation
Source: PLoS One. 2021 Apr 29;16(4):e0236684. doi: 10.1371/journal.pone.0236684 (PMC8084136; doi:10.1371/journal.pone.0236684)
Supplement: S2 Fig — (A) Example heat map matrices of the pairwise stimulus-evoked correlation coefficient between a population of 74 neurons within the cFL somatosensory cortex of one example animal at 160μm depth for 0.1s 100Hz, 0.05s 200Hz, 1s 3Hz, and 1s 10Hz stimuli. Structural similarity comparison of the maps depicts greater map similarity for within stimulus trial conditions than between stimulus trial comparisons (B). A significant effect of stimulus is observed in the percent of neurons responding (D) and in the neuron decay tau (F), but not in the neuron response amplitude (C) or the neuron AUC (E). (G) Color coded quantification chart displaying the percentage overlap between neuron populations responsive to each stimulus quantified for all animals (N = 10). *p < 0.05; **p < 0.01; ***p < 0.001. (DOCX) [file pone.0236684.s002.docx]

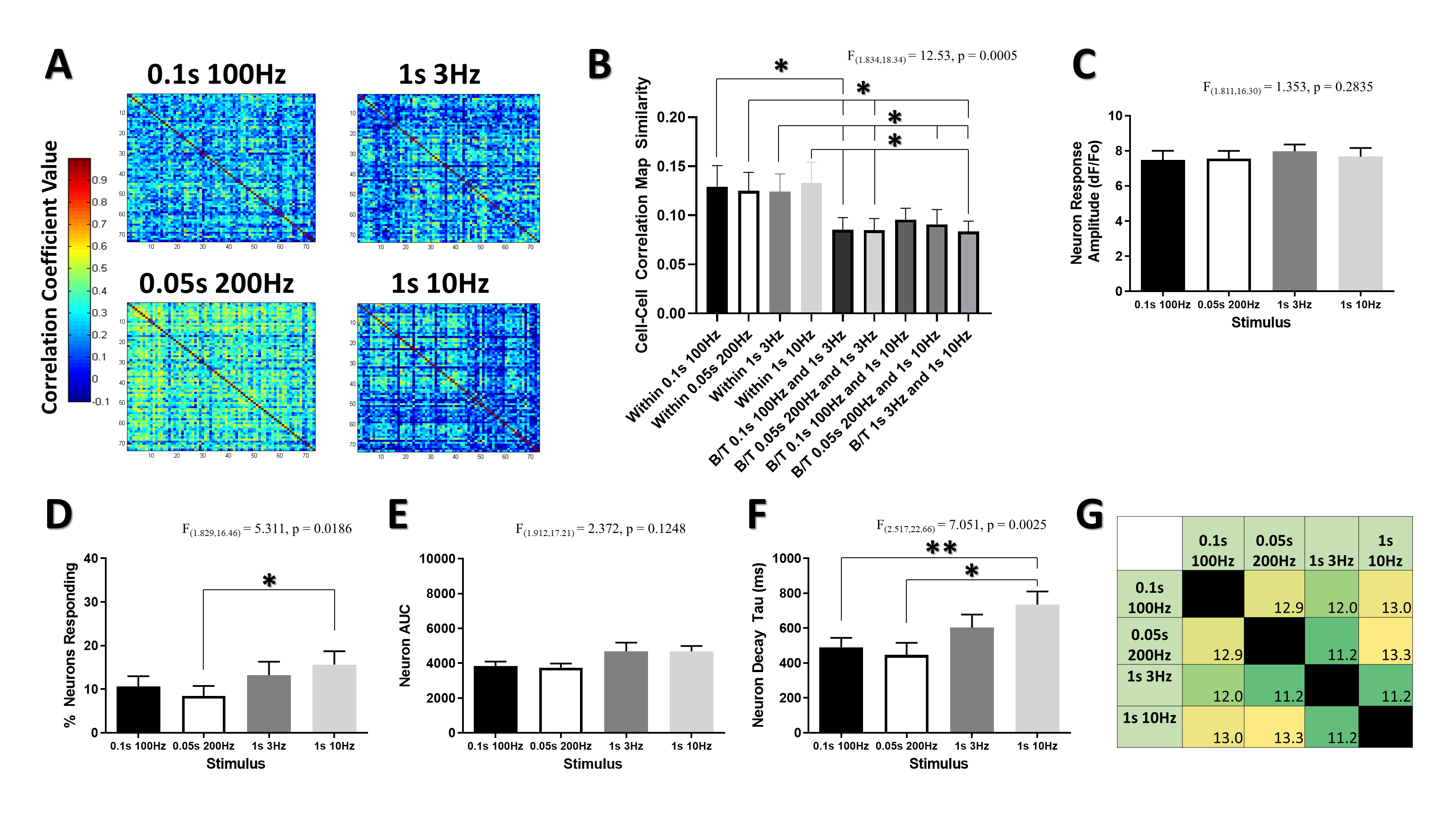


**Fig S2. Short duration, high frequency stimuli lead to differential cortical activity from long duration, low frequency stimuli.** (***A***) Example heat map matrices of the pairwise stimulus-evoked correlation coefficient between a population of 74 neurons within the cFL somatosensory cortex of one example animal at 160μm depth for 0.1s 100Hz, 0.05s 200Hz, 1s 3Hz, and 1s 10Hz stimuli. Structural similarity comparison of the maps depicts greater map similarity for within stimulus trial conditions than between stimulus trial comparisons (***B***). A significant effect of stimulus is observed in the percent of neurons responding (***D***) and in the neuron decay tau (***F***), but not in the neuron response amplitude (***C***) or the neuron AUC (***E***). (***G***) Color coded quantification chart displaying the percentage overlap between neuron populations responsive to each stimulus quantified for all animals (N=10). *p < 0.05; **p < 0.01; ***p < 0.001
