# Supplementary material for: Distinct patterns of activity in individual cortical neurons and local networks in primary somatosensory cortex of mice evoked by square-wave mechanical limb stimulation
Source: PLoS One. 2021 Apr 29;16(4):e0236684. doi: 10.1371/journal.pone.0236684 (PMC8084136; doi:10.1371/journal.pone.0236684)
Supplement: Attachment — Submitted filename: Reply to Reviewers March 2021 For Submission.docx [file pone.0236684.s004.docx]

**Reviewer #1: The authors have done an unsatisfactory job in addressing the reviewers’ concerns and comments. Many of the questions from the initial revision were not answered or not properly addressed. These are detailed here below.**

**Moreover, the authors now state that the main contribution of their work is that the “characterization of the distinct patterns of neural activity elicited by such stimuli is of importance as these forms of squarewave stimulation are used in multiple fields outside of the study of sensory coding, such as in the observation of cortical recovery from damage or disease.”**

**It however remains unclear what exact implication these results can have on studies of cortical recovery from disease or other studies using similar sensory stimulation. The authors must do a better job arguing this point, beyond simple hand-waving. Otherwise, I really do not see the point of this manuscript and its results.**

We thank the reviewer for his thorough review of the manuscript and suggestions for amendments, and apologize if our previous revisions were not sufficient. We have amended the introduction to further highlight the implications of our study for understanding somatosensory representations of mechanical square-wave stimuli under anesthetized conditions as follows:

Investigations of how the somatosensory cortex responds to artificial forms of stimulation, and to what extent patterns of regional and cellular activity can distinguish between distinct stimuli with overlapping characteristics, is important in interpreting studies that use such stimuli to elicit cortical responses in the healthy brain, or as a measure of cortical excitability and plasticity during disease or after injury [1–5]. While square-wave and sinusoidal patterns of mechanical limb stimulation have been used in studies after cortical injury, and those studies identified deficits in the amplitude and fidelity of evoked activity after damage to the somatosensory cortex (for example see [1,2,4,6]), a more detailed investigation across multiple frequencies and intensities of stimulation has not been performed with mechanical square-wave stimuli. Mechanical square-wave stimulation allows precise control of the fundamental frequency (as with sinusoidal stimulation) but includes harmonic frequencies. These harmonics may overlap between different stimuli, and it may, therefore, be more challenging for the somatosensory system to distinguish between these stimuli. Whereas considerable literature exists for somatosensation within the healthy brain in non-human primates (for review, see [7,8]), and the sensory-evoked response properties of the barrel cortex of rodents has been extensively studied [9,10], literature on the sensory evoked response properties of limb associated somatosensory system of rodents is more limited.

**Abstract:**

**- Lines 36-39: I have trouble understanding the difference between “process dissimilar stimuli with distinct patterns of activity” and “represent distinct […] stimuli with unique patterns of activity”.**

We have amended the abstract as follows:

Artificial forms of mechanical limb stimulation are used within multiple fields of study to determine the level of cortical excitability and to map the trajectory of neuronal recovery from cortical damage or disease. Square-wave mechanical or electrical stimuli are often used in these studies, but a characterization of sensory-evoked response properties to square-waves with distinct fundamental frequencies but overlapping harmonics has not been performed. To distinguish between somatic stimuli, the primary somatosensory cortex must be able to represent distinct stimuli with unique patterns of activity, even if they have overlapping features. Thus, mechanical square-wave stimulation was used in conjunction with regional and cellular imaging to examine regional and cellular response properties evoked by different frequencies of stimulation. Flavoprotein autofluorescence imaging was used to map the somatosensory cortex of anaesthetized C57BL/6 mice, and *in vivo* two-photon Ca^2+^ imaging was used to define patterns of neuronal activation during mechanical square-wave stimulation of the contralateral forelimb or hindlimb at various frequencies (3, 10, 100, 200, and 300 Hz). The data revealed that neurons within the limb associated somatosensory cortex responding to various frequencies of square-wave stimuli exhibit stimulus-specific patterns of activity. Subsets of neurons were found to have sensory-evoked activity that is either primarily responsive to single stimulus frequencies or broadly responsive to multiple frequencies of limb stimulation. High frequency stimuli were shown to elicit more population activity, with a greater percentage of the population responding and greater percentage of cells with high amplitude responses. Stimulus-evoked cell-cell correlations within these neuronal networks varied as a function of frequency of stimulation, such that each stimulus elicited a distinct pattern that was more consistent across multiple trials of the same stimulus compared to trials at different frequencies of stimulation. The variation in cortical response to different square-wave stimuli can thus be represented by the population pattern of supra-threshold Ca^2+^ transients, the magnitude and temporal properties of the evoked activity, and the structure of the stimulus-evoked correlation between neurons.

**Introduction:**

**- Lines 58-59: Can the authors provide references to studies using square-wave pulses “as measures of cortical excitability and plastic changes during disease or after injury”?**

We have amended the introduction as follows:

Investigations of how the somatosensory cortex responds to artificial forms of stimulation, and to what extent patterns of regional and cellular activity can distinguish between distinct stimuli with overlapping characteristics, is important in interpreting studies that use such stimuli to elicit cortical responses in the healthy brain, or as a measure of cortical excitability and plasticity during disease or after injury [1–5]. While square-wave and sinusoidal patterns of mechanical limb stimulation have been used in studies after cortical injury, and those studies identified deficits in the amplitude and fidelity of evoked activity after damage to the somatosensory cortex (for example see [1,2,4,6]), a more detailed investigation across multiple frequencies and intensities of stimulation has not been performed with mechanical square-wave stimuli. Mechanical square-wave stimulation allows precise control of the fundamental frequency (as with sinusoidal stimulation) but includes harmonic frequencies. These harmonics may overlap between different stimuli, and it may, therefore, be more challenging for the somatosensory system to distinguish between these stimuli. Whereas considerable literature exists for somatosensation within the healthy brain in non-human primates (for review, see [6,7]), and the sensory-evoked response properties of the barrel cortex of rodents has been extensively studied [9,10], literature on the sensory evoked response properties of limb associated somatosensory system of rodents is more limited.

**- Lines 60-61: This sentence should be nuanced. Literature on the somatosensory system of rodents is quite extensive if taking into account the vibrissae system.**

We have amended the manuscript as follows to make it more nuanced:

Investigations of how the somatosensory cortex responds to artificial forms of stimulation, and to what extent patterns of regional and cellular activity can distinguish between distinct stimuli with overlapping characteristics, is important in interpreting studies that use such stimuli to elicit cortical responses in the healthy brain, or as a measure of cortical excitability and plasticity during disease or after injury [1–5]. While square-wave and sinusoidal patterns of mechanical limb stimulation have been used in studies after cortical injury, and those studies identified deficits in the amplitude and fidelity of evoked activity after damage to the somatosensory cortex (for example see [1,2,4,6]), a more detailed investigation across multiple frequencies and intensities of stimulation has not been performed with mechanical square-wave stimuli. Mechanical square-wave stimulation allows precise control of the fundamental frequency (as with sinusoidal stimulation) but includes harmonic frequencies. These harmonics may overlap between different stimuli, and it may, therefore, be more challenging for the somatosensory system to distinguish between these stimuli. Whereas considerable literature exists for somatosensation within the healthy brain in non-human primates (for review, see [6,7]), and the sensory-evoked response properties of the barrel cortex of rodents has been extensively studied [9,10], literature on the sensory evoked response properties of limb associated somatosensory system of rodents is more limited.

**- Lines 61-62: Rodents are certainly not the “most prominent animal model used to study reaching and upper limb use and disability”. Sensorimotor studies on the rodent upper limb system have emerged only relatively recently. This system has been traditionally and most prominently studied in non-human primates. Also the reference that is cited here ([3]) has nothing to do with upper limb use and reaching, it instead provides a general comparison between rats and mice as models in biomedical research.**

We agree with the reviewer on this point, and apologize for the error in citation. We have amended the introduction as follows:

Investigations of how the somatosensory cortex responds to artificial forms of stimulation, and to what extent patterns of regional and cellular activity can distinguish between distinct stimuli with overlapping characteristics, is important in interpreting studies that use such stimuli to elicit cortical responses in the healthy brain, or as a measure of cortical excitability and plasticity during disease or after injury [1–5]. While square-wave and sinusoidal patterns of mechanical limb stimulation have been used in studies after cortical injury, and those studies identified deficits in the amplitude and fidelity of evoked activity after damage to the somatosensory cortex (for example see [1,2,4,6]), a more detailed investigation across multiple frequencies and intensities of stimulation has not been performed with mechanical square-wave stimuli. Mechanical square-wave stimulation allows precise control of the fundamental frequency (as with sinusoidal stimulation) but includes harmonic frequencies. These harmonics may overlap between different stimuli, and it may, therefore, be more challenging for the somatosensory system to distinguish between these stimuli. Whereas considerable literature exists for somatosensation within the healthy brain in non-human primates (for review, see [6,7]), and the sensory-evoked response properties of the barrel cortex of rodents has been extensively studied [9,10], literature on the sensory evoked response properties of limb associated somatosensory system of rodents is more limited. Given that rodents are a prominent animal model used to study recovery of reaching and upper limb use after central nervous system damage or disability [8,9], it is important to understand how different forms of somatic and movement related stimuli used in rodent research are processed in the rodent somatosensory cortex. Most current research on the limb-associated somatosensory cortex in rodents utilize artificial forms of mechanical[1,10–12] or electrical stimuli[13,14] to assess sensory-evoked responses using electrophysiology of individual cells or aggregate responses from large cortical regions.

**- Lines 67-69: Why is this point relevant here? Do the authors work towards solving this issue in their study?**

We agree that these comments were not relevant to the proposed work, and have removed these comments in the revised manuscript.

**- Lines 96-98: I find this description too vague. Consider rewriting by more specifically stating the actual findings.**

We have amended the section of the introduction as follows:

Calcium imaging has been used as a method for studying activity within regional cortical networks[1,21–24]. Here, Ca^2+^ imaging was used to investigate how the limb-associated somatosensory cortex represents different frequencies of square-wave limb oscillation, and if differential patterns of activity in somatosensory neurons and local networks could be detected within the Ca^2+^ response. Flavoprotein autofluorescence imaging[25–29] was first used to identify limb-associated regions of cortical activation during mechanical forelimb or hindlimb stimulation delivered with piezoelectric actuators[1,30] (Fig 1a). *In vivo* two-photon Ca^2+^ imaging was used to optically record the response properties of individual neurons and local neuronal networks within these limb-associated somatosensory regions during multiple frequencies of contralateral forelimb (cFL) or contralateral hindlimb (cHL) oscillation (Fig 1b-d). Our data shows that the magnitude of the neuronal population response in somatosensory cortex is non-linearly related to the frequency of mechanical limb stimulation. High frequency 100Hz, 200Hz and 300Hz stimuli were found to elicit more responding neurons, a greater response strength, and higher average cross-correlation between neurons than lower frequency 3Hz and 10Hz stimuli. Whereas average population response magnitude within the low frequency and high frequency groups was not a strong differentiator of how populations represented stimuli within these groups, the pattern of responsive neurons within the local neuronal network and differences observed in the cross-correlation maps for populations responding to these stimuli clearly differentiated between square-wave stimuli within and between stimulus groups.

**Methods**

**- Lines 142-143: Can the authors provide the coordinates relative to bregma of the identified cFL and cHL areas? This was asked in the previous revision but was not answered!**

Please note that a coordinate based system was not used to define the exact position of the cFL and cHL, as the stereotaxic coordinates of these regions can vary from strain to strain, from colony to colony within a particular strain, and even to some degree from mouse to mouse within a colony. An area on the skull surface with coordinates +2mm to -2mm anterior-posterior, and +1mm to +5mm lateral to bregma was thinned, then flavoprotein IOS imaging performed using a low magnification (2.5X) objective capturing the entire thinned area. Flavoprotein IOS denoted the precisely mapped cortical areas responsive to the cFL or cHL, respectively. A craniotomy of approximately 3x3mm was then centered over the approximate centre of mass of the cFL/cHL region. The cFL and cHL regions mapped via flavoprotein IOS were used for injection of the OGB-1 indicator. These maps were also used to determine the precise locations for Ca^2+^ imaging within these regions. Approximate center of mass coordinates of the cHL was 2mm lateral, -1mm posterior. Approximate coordinates for the cFL was +1mm anterior, +2.5mm lateral from bregma. We have further amended the methods with the following to further clarify:

A 4 x 4 mm region of the skull overlying the right hemisphere somatosensory region was thinned to 25-50% of original thickness using a high-speed dental drill (~1-5mm lateral, +2 to -2 mm posterior to bregma). This thinned region was covered with 1.3% low-melt agarose dissolved in artificial CSF (ACSF) at 37^o^C, then covered with a 3mm glass coverslip. Flavoprotein autofluorescence (FA) imaging was performed through this thin skull preparation before *in vivo* Ca^2+^ imaging to determine somatosensory limb regions on the cortex. These mapped regions were later used for OGB-1 indicator injection and for determination of imaging window coordinates for Ca^2+^ imaging following the FA mapping.

**- In the initial review, I asked the authors to provide information about the actual amplitude of vibrations produced at each frequency. The authors replied that they amended the methods with the following information: “During stimulation, the entire limb underwent an oscillation with the following peak-peak amplitudes by frequency: 280um (3 & 10Hz), 335um (100Hz), 220um (200Hz), 170um (300Hz).”.**

**No detail is however given about how these amplitudes were measured! The authors mention that they “imaged the limb oscillations” with no additional technical detail. A 300 Hz oscillation would require a minimum 600 fps frame rate to capture the movement amplitude. Also, the following questions were not answered: At what sampling rate were the oscillations produced? How was the stimulus integrity assured? Was it consistent across several repetitions?**

The movement of the limb was imaged using a Dalsa Pantera 1M60 camera mounted on a Leica SP5 microscope using a 2.5X objective. The limb was attached to the stimulator and placed on a black background to increase contrast of the limb relative to the background. Three different imaging frame rates and resolutions were recorded for each frequency of limb stimulus based on the limits imposed by the camera hardware: 58fps at 1024x1024, 100fps at 512x512 and 157fps at 256x256. As all of these imaging frame rates were undersampling relative to the Nyquist criterion for the 300Hz stimuli, we employed several measures to get an index of the peak-to-peak amplitude of the limb oscillation as follows. For all imaging framerates, we recorded extended video (upwards of 5 minutes per recording), then used max intensity projections to determine the maximal bending amplitude of the stimulus at each stimulus frequency. To confirm this method, we compared the max projection measurements and frame-per-frame measurements for low frequency stimuli and found them to be equal. Peak-to-peak deviation measurements from all three imaging framerates at all frequencies were compared and found not to be significantly different. To determine if the peak-to-peak bending amplitude was consistent across multiple stimulus trials, we repeated the video recordings multiple times and separately analyzed each trial. Bending amplitudes were consistent across trials of the same stimulus frequency. Bending actuators were driven by an A-M Systems Model 2100 stimulator, an analog stimulator with 1 microsecond timing (250 ns jitter). These methods are not included in the methods section, but could be added if warranted in the reviewer’s opinion.

**Results**

**Fig. 2B,C: The traces correspond to averages of 8 trials (no information about trail-to-trial variability is given). Can the authors also show the individual Ca traces from every trial? What do the polygons in the bottom panels represent (they are not mentioned in the figure legend)? Also, the labels on these polygons are tiny and thus unreadable.**

Figures have been revised with individual traces from every trial. Plots are revised and figure legend edited to mention that the plots demonstrate the peak Ca^2+^ response amplitude of the cell to each of the stimuli.

**Lines 268-270: This is entirely qualitative. Can the authors demonstrate statistically that the responses of these example neurons show stimulus specificity?**

We agree with the reviewer that these neurons are not showing specificity, only variance in their Ca^2+^ response magnitude to the different stimuli and preference for certain stimuli over others. The plots below the Ca^2+^ traces have been edited to make them more visible. These show the quantified response magnitude of the above neuron to each of the stimuli. We have amended the text to read as follows:

Fig 2b,c depict representative neurons selected from the imaging plane shown in A that display different magnitudes in their Ca^2+^ response to the different stimuli. Notably, the neuron in Fig 2b is more responsive to high frequency 100, 200, and 300Hz stimuli, whereas the neuron in Fig 2c exhibits a mean response that is transient and most strongly activated by 10Hz stimulation.

**Fig.2: According to the activity maps in Fig.2A and 2D, a relatively large number of neurons is driven by the vibrotactile stimulation. This is in contradiction to what has previously been reported for L2/3 of the somatosensory cortex. Even in awake behaving mice, evoked activity by tactile stimulation remains very sparse. O’Connor et al. (Neuron, 2010) found that “A sparse subset of L2/3 neurons showed robust fluorescence transients […] high event rates were seen in only a small subset of neurons, with the majority showing low, near zero, event rates” in the vibrissae S1. Prsa et al. (Nature, 2019) reported 1,285 responsive neurons from 75 fields of view in the forelimb S1, yielding ca. 17 neurons per field of view. The authors found a much larger number of responding neurons under anaesthesia (Fig.2A,D). Can the authors quantify these numbers and how do they explain the discrepancy with previous studies?**

Our results demonstrated approximately 15% of cells exhibiting a significant response to 3Hz or 10Hz stimuli, and approximately 30% of the population responding for each of the 100, 200, or 300Hz stimuli. This is quantified in Fig. 2E. These results are consistent with Hayashi et al., 2018 that performed a similar set of experiments using a vibrotactile stimulus applied to the limb of the mouse, also under anesthetized conditions. Note that the activity maps in Fig. 2A display not only deltaF/Fo within neurons, but also within the neuropil surrounding neurons. We have amended the results with the following:

Notably, high frequency 100 & 200Hz stimuli displayed a significantly greater percentage of above threshold responses relative to 3 & 10Hz stimuli. Consistent with previous research by Hayashi et al., 2018, approximately 15% of neurons exhibited a significant response for the 3Hz or 10Hz stimuli, and approximately 30% of the population exhibited a significant response for each of the 100, 200, or 300Hz stimuli (Fig 2e). The majority of neurons with above threshold Ca^2+^ responses were responding to multiple stimuli, with approximately 32.7 ± 4.2% selective to a single stimulus, 33.2 ± 1.8% activated by two stimuli, and 34.1 ± 3.8% activated by three or more stimuli (Fig 2f).

**The authors also avoided addressing the following comment from the previous revision:**

**“Why was such a high amplitude chosen? Previous studies in rodents used an order of magnitude smaller amplitudes (in the um range) which evoked highly reliable responses and is already well beyond their perceptual thresholds. It therefore seems highly plausible that the 1 mm oscillations evoked highly saturated neural responses, which are definitely not in the natural perceptual range of mice.”**

**and**

**The following question from the previous revision was not answered: “Page 12, lines 266-267: could this be due to saturated Ca responses?”**

The stimulator moved the entire limb and was not directed to a focal location on the glabrous skin. The large amplitude stimuli were chosen to elicit strong activity in proprioceptive receptor populations of the limb in addition to cutaneous receptor activation at the site of stimulator attachment. We agree with the reviewer that the stimuli are not naturalistic, however it is speculation on whether the neural responses were saturated or not. We have amended the discussion with the following text to address this possibility:

This study did not attempt to directly examine the contribution of specific mechanoreceptor populations in the limbs to the afferent signal generated by our square-wave stimuli and their corresponding cortical responses. While it is not possible to give specific predictions on how strongly each modality was represented within the afferent response and cortical activity, peripheral mechanoreceptors are known to have varied activation thresholds at a range of frequencies[75,76], and the overall pattern of evoked activity would therefore reflect mixed afferent signals generated by the stimuli in this study. Saturation of the calcium response for high frequency limb oscillation could potentially contribute to similarities in the percent of neurons responding (Fig 2e) and the average population response magnitude (Fig 3) to our high frequency stimuli. However, distinct patterns of cellular responses for neurons responsive to each of the high frequency stimuli (Fig 2g), and differences observed in the correlational map structure between different high frequency stimuli (Figs 5,6), suggest that the calcium response maintained sufficient fidelity to record distinct representations of stimulus frequency. Future studies could make use of measurement methods with higher temporal fidelity and transgenic animals expressing channelrhodopsins in genetically-identified modality specific afferents to further examine the contribution of particular modalities to the cortical response[62].

**The following question from the previous revision was not answered:**

**“A threshold criterion was used to identify responsive neurons. Was this analysis done on the average traces or on every single trial? The authors claim that this procedure was “optimal for separating responsive neurons from noise”, but based on what criteria?” The authors still state in the manuscript that their criterion is “optimal”. How is optimality assessed? Compared to what?**

The threshold criterion was chosen as an effective method to identify signal from noise in averaged traces consistent with previous studies (e.g. Roth et al., 2012, Winship et al., 2007,2008). We have further amended the methods as follows to highlight how this was assessed:

Due to the large number of signal traces that required analysis (over 40,000 traces; over 6000 neurons; 10 mice), a range of threshold criteria, based on previous research [1,23,32–34], were tested to differentiate responsive neuron Ca^2+^ transients from fluorescent noise. This range of threshold criteria was tested against manual annotation of Ca^2+^ responses gathered from multiple observers for a small subset of the experimental dataset from multiple animals. Positive identification of a Ca^2+^ transient from noise within the manual annotation was based on the expected Ca^2+^ transient waveform demonstrating fast rise on the leading edge of the fluorescence, and a slow decay back to baseline. The threshold criteria found to most effectively select Ca^2+^ transient waveforms that met this expected waveform shape was selected for identification of Ca^2+^ transients across all animals. A threshold criteria requiring the Ca^2+^ fluorescence of the cell, averaged from 8 trials, to increase by 3X the standard deviation of the baseline period ΔF/F_o_ (baseline defined as 1s before stimulus onset), and remain above this criteria for 160ms (4 successive frames), was used to differentiate the Ca^2+^ transient waveform of a response of the neuron from random noise fluctuations.

**The authors gave the following answer to the question regarding the analysis of the Ca transient decay:**

**“If we were to speculate, a longer decay tau for the population response to high frequency stimuli may indicate that the neuronal population takes longer to desensitize in its firing rate over the period of the 1 second stimulation leading to a longer calcium transient. This however would have to be shown in future studies measuring the desensitization of spiking activity using electrical neurophysiology instead of calcium imaging due to the slow temporal dynamics of calcium imaging.”**

**I agree with the authors that this is highly speculative and also still do not understand why it is relevant to analyse the decay. I would suggest removing this analysis from the manuscript.**

We agree that this is speculative, and accordingly have removed the decay analysis from the manuscript as suggested.

**The authors answered the following in the previous revision:**

**“We agree with the reviewers interpretation; it is likely that the stronger and more frequent responses to higher frequency stimuli directly lead to the higher average crosscorrelation measured.” The authors should therefore clearly state this in their manuscript.**

We have amended the discussion with the following:

A greater average cross-correlation for high frequency stimuli was observed (Fig 4e), potentially due to stronger and more frequent responses within the neural population to these high frequency stimuli.
