# Supplementary material for: Distinct patterns of activity in individual cortical neurons and local networks in primary somatosensory cortex of mice evoked by square-wave mechanical limb stimulation
Source: PLoS One. 2021 Apr 29;16(4):e0236684. doi: 10.1371/journal.pone.0236684 (PMC8084136; doi:10.1371/journal.pone.0236684)
Supplement: Attachment — Submitted filename: Reply to Reviewers April 2021.docx [file pone.0236684.s005.docx]

**Reviewer #1: Two final comments:
- I would suggest including the description of how limb movement amplitudes were measured in the Methods section.
- I suggest adding reference 23 to the sentence: " ... and transgenic animals expressing channelrhodopsins in genetically-identified modality specific afferents to further examine the contribution of particular modalities to the cortical response[62]. "**

We thank the reviewer for their final comments for our manuscript. We have added reference 23 to the sentence and have added the following description of how limb movement amplitudes were measured to the methods section:

During stimulation, the entire limb underwent an oscillation with the following peak-peak amplitudes by frequency based on the limb weight loaded electromechanical properties of the bending actuator: 280um (3 & 10Hz), 335um (100Hz), 220um (200Hz), 170um (300Hz). To determine these peak-peak oscillation amplitudes, the movement of the limb was imaged using a Dalsa Pantera 1M60 camera mounted on a Leica SP5 microscope using a 2.5X objective. The limb was attached to the stimulator and placed on a black background to increase contrast of the limb relative to the background. Bending actuators were driven by an A-M Systems Model 2100 stimulator, an analog stimulator with 1 microsecond timing (250 ns jitter). Three different imaging frame rates and resolutions were recorded for each frequency of limb stimulus based on the limits imposed by the camera hardware: 58fps at 1024x1024 (17.24ms exposure), 100fps at 512x512 (10ms exposure) and 157fps at 256x256 (6.37ms exposure). As all of these imaging frame rates were undersampling relative to the Nyquist criterion for the 300Hz stimuli, we employed several measures to get an index of the peak-to-peak amplitude of the limb oscillation as follows. For all imaging framerates, we recorded extended video (approximately 5 minutes per recording), then used max intensity projections to determine the maximal bending amplitude of the stimulus at each stimulus frequency. To confirm this method, we compared the max projection measurements and frame-per-frame measurements for low frequency stimuli and found them to be equal. Peak-to-peak deviation measurements from all three imaging framerates at all frequencies were compared and found not to be significantly different. To determine if the peak-to-peak bending amplitude was consistent across multiple stimulus trials, we repeated the video recordings multiple times and separately analyzed each trial. Bending amplitudes were consistent across trials of the same stimulus frequency.
